# “The heat really troubles us”: community perspectives on a cool roof intervention, Umlazi, KwaZulu-Natal, South Africa

**DOI:** 10.3389/fclim.2026.1873846

**Published:** 2026-07-26

**Authors:** Saloshni Naidoo, Patrick Nyamaruze, Nisha Nadesan-Reddy, Nompumelelo Nzimande, Isabel Madzorera, Cheng He, Till Bärnighausen, Wafaie Fawzi

**Affiliations:** 1 Discipline of Public Health Medicine, School of Medicine, University of KwaZulu-Natal, Durban, South Africa; 2 Discipline of Development and Population Studies, School of Social Sciences, University of KwaZulu-Natal, Durban, South Africa; 3 Division of Community Health Sciences, School of Public Health, University of California, Berkeley, Berkeley, CA, United States; 4 Department of Global Health and Population, Harvard T.H. Chan School of Public Health, Boston, MA, United States; 5 Heidelberg Institute of Global Health (HIGH), Faculty of Medicine and University Hospital, Heidelberg University, Heidelberg, Germany; 6 Africa Health Research Institute (AHRI), KwaZulu-Natal, South Africa; 7 Harvard Center for Population and Development Studies, Cambridge, MA, United States; 8 Department of Epidemiology, Harvard T.H. Chan School of Public Health, Boston, MA, United States; 9 Department of Nutrition, Harvard T.H. Chan School of Public Health, Boston, MA, United States

**Keywords:** community perceptions, cool-roof intervention, heat, peri-urban, sustainability

## Abstract

**Introduction:**

Climate change disproportionately affects communities experiencing social and environmental vulnerabilities. In South Africa, rising temperatures pose a significant risk to peri-urban communities. This pilot study explored community perceptions and attitudes toward the “cool roof” passive cooling technology for homes, as a climate adaptation strategy in Umlazi, South Africa.

**Method:**

Trained facilitators conducted focus group discussions with advisory board members from the Umlazi Health and Demographic Surveillance Site (USINGA) a peri-urban demographic surveillance node located in Umlazi, KwaZulu-Natal, South Africa, using a semi-structured guide and visual aids of cool roofs, to explore perceptions and attitudes toward cool roof interventions. Discussions were recorded, transcribed and analyzed using inductive thematic analysis following Braun and Clarke’s approach.

**Results:**

The emerging themes were: limited awareness and knowledge of cool roofs as a heat adaptation strategy, perceived benefits and acceptance, concerns and reservations, affordability and equity, design preferences, as well as the importance of partnerships and community engagement. Participants recognized the potential of cool roofs in reducing indoor heat, improving comfort, reducing extreme fatigue, and benefiting vulnerable populations; they viewed the intervention as timely and necessary. However, concerns were raised that some older people might believe that white roofs attract death, and some may indulge in the conspiracy theory that the coating is associated with disease. Participants questioned the long-term effectiveness of cool roofs and their compatibility with existing informal housing structures lacking traditional roofs. They felt that the current cost of paint may make the intervention inaccessible to those most in need of it, thereby limiting equitable distribution, as most people were unemployed. Furthermore, they believed that if cool roofs lacked long-term durability and required constant maintenance, people were unlikely to accept the intervention.

**Conclusion:**

While overall perceptions were positive, engaging and educating the community through transparent communication and information on cool roof interventions will address concerns and encourage the adoption of these interventions. From a policy and practice perspective, government programs on climate resilience must be culturally sensitive and responsive to local beliefs while prioritizing vulnerable populations and being inclusive during climate adaptation planning. Further research is needed to inform the development of acceptable and scalable implementation strategies in peri-urban settings.

## Introduction

Climate change is resulting in temperature increases globally, and this trend is also evident in Africa (Food and Agriculture Organization of the United Nations, 2025). Southern Africa has experienced a steady increase in temperatures over the past few years, including South Africa, with the potential for adverse health impacts (Intergovernmental Panel on Climate Change, 2021; Intergovernmental Panel on Climate Change, 2022; Academy of Science of South Africa, 2026). It is anticipated that most areas in South Africa will experience increases in the intensity, frequency and duration of high temperatures in the future (McBride et al., 2025).

The impacts of temperature extremes and heat stress on human health have been documented and include acute and chronic illnesses that affect health directly and indirectly (Xu et al., 2025). Acute direct health impacts of heat stress typically manifest in heat exhaustion and heat stroke, and an increase in heat-related morbidity and mortality (Bukhari, 2023). Additionally, chronic heat-related illnesses such as cardiac disease, respiratory disease, and adverse pregnancy outcomes are also often observed consequences (Liu et al., 2022; Zhu et al., 2025; Chersich et al., 2020).

Throughout Africa, and in particular in southern Africa, the most vulnerable communities bear the brunt of the impact of temperature increases (Tamoffo, 2025; Kunda et al., 2024). These communities are typically located in peri-urban or rural areas, with a high burden of social, environmental, and economic challenges and with limited capacity and resources to mitigate heat exposures. The actual structure of houses, which often includes a lack of ventilation, tin-roofs and the absence of cooling solutions, poor urban planning, a lack of vegetation, and limited access to water, increases the risk of heat-related illnesses in these settings (Parker et al., 2025; Tusting et al., 2019).

Therefore, there is a need for interventions to reduce surface and indoor temperatures in community settings, which are being tested in several parts of the world. These interventions include green roofs and walls, cool roofs, and modified asphalt material pavements (Das et al., 2025). Several systematic reviews have reported reductions in surface and indoor temperature in the presence of various temperature reduction strategies (Das et al., 2025; Jain, 2024; Johar et al., 2025). A systematic meta-analysis reported significant reductions of 1.5 °C to 3.5 °C in indoor temperatures when using green roofs, green walls, and cooling reflective paint on roofs compared to standard roofs and walls (Das et al., 2025). Similarly, research in multiple countries has shown a reduction in surface and indoor temperatures when using cooling reflective roof paints. Wai et al. (2025) in their study of six different cool roof paints in Melbourne, Australia, reported surface temperature reductions of between 8.7 °C and up to 34.2 °C. A study in rural and peri-urban India evaluated cool roof impacts on indoor temperatures and reported an average and peak reduction in indoor temperatures of 2.1 °C and 5.0 °C, respectively (Garg et al., 2016). Findings from Burkina Faso show that cool-roof coatings reduce indoor temperature up to 2.7 °C, which may lead to possible health benefits in the study population (Bunker et al., 2024).

As these cooling strategies are being tested and introduced in various settings, understanding community perceptions and attitudes toward such interventions is important. A qualitative study on the acceptability of cool roofs in Nouna, Burkina Faso, found that the community would accept cool roof interventions if the roofs were effective in reducing temperatures, of good quality, affordable, long-lasting and locally produced (Barnighausen et al., 2025). In South Africa, while still in the early days, there are cool-roof initiatives underway. A study conducted in informal dwellings in Lenasia, Gauteng, reports changes in mean indoor temperatures of 2.2 °C to 4.3 °C in dwellings with the cool paint intervention when compared to dwellings without the intervention (Kimemia et al., 2020). The South African Energy Development Institute (SANEDI) implemented a cool-roof initiative in the Northern Cape Province in approximately 425 houses, reporting decreases in indoor daytime temperature between 8 °C and 10 °C (South African Cool Surfaces Association, 2024). A modelling and field study in an informal settlement in Tshwane, Gauteng, found that cool roof paints reduced excessive heat stress conditions by approximately 42% to 63% in houses with corrugated iron roofs (Hugo, 2023). However, to the best of our knowledge, no study has been published on community perceptions and attitudes towards cool-roof interventions in peri-urban settings in South Africa. Hence, understanding community perceptions and attitudes toward the intervention in our study is valuable for policymakers and the government as they embrace the intervention as a climate resilience strategy.

KwaZulu-Natal (KZN) is one of South Africa’s nine provinces, situated on the eastern seaboard. This part of the country experiences extremely high temperatures for a significant portion of the year, with a high risk of heat-related illnesses. While there is limited heat-related health outcomes research published from the province suggests that extreme heat exposure may result in an increase in miscarriages amongst women (Moodley et al., 2024). Hence, it is important to consider heat-reduction strategies for KZN communities.

As a first step in piloting a cool roof intervention in Umlazi, KZN, we conducted a qualitative study piloting a tool to explore community awareness and knowledge, perceptions, and attitudes toward the concept of cool roofs. The study also examined perceptions regarding participation and community engagement in guiding the implementation of cool roofs.

## Methods

### Study design and setting

This was a qualitative explorative study conducted in the Umlazi Health and Demographic Surveillance System (USINGA) a peri-urban demographic surveillance node located in Umlazi, KwaZulu-Natal, South Africa. The surveillance node collects longitudinal population data in a circumscribed population of Umlazi (Umlazi Surveillance Initiative to Nurture Grassroots Action, n.d.). Umlazi is a peri-urban suburb located in the largest municipality (eThekwini) in KZN with a population of approximately 400,000 people (Statistics South Africa, 2018). This is a suburb with a largely working-class population experiencing several socio-economic challenges, including high levels of unemployment (Statistics South Africa, 2018). Households consist of a mixture of formal and informal structures. The study location, eThekwini, has experienced the impact of adverse environmental events, including floods and heat waves, between 2022 and 2026 (Nemakonde, 2025; Jacobs, 2026).

### Study population

The study population (*n* = 20) comprised representatives from the USINGA community. The participants constitute the USINGA Community Advisory Board (CAB). They are key role players in the Umlazi community and were chosen by the residents of Umlazi to represent their interests in USINGA. The involvement of CAB members in the first stage of the project was because they are from the community and are representative of the community. Since they are CAB members and have been involved with the implementation of USINGA they may be more knowledgeable than community members, which could have led to information bias in our study.

### Data collection

Data collection was through focus group discussions (FGDs), which followed a semi-structured guide designed to explore community participants’ perceptions and attitudes towards cooling roof interventions. Two FGDs, which lasted on average for 2 hours, were conducted. The FGDs were conducted by a team of facilitators who are part of the community and work as part of USINGA. Each FGD was conducted in isiZulu and led by a trained facilitator experienced in conducting FGDs.

The cool roof intervention, which we propose using was explained to the participants. The cool roof intervention will comprise a paint coating applied to the external surface of the roof. The selected paint will have a minimum solar reflection of at least 75%, a thermal emittance of at least 75% (South African Cool Surfaces Association, 2024), and can be easily applied to a variety of roof types such as corrugated iron, asbestos, and concrete tiles. Homeowners will be able to apply the paint, which requires washing annually to maintain the surface. Two coats of undiluted paint, following surface preparation, can be applied 4 hours apart with an expected durability of 10 years (Rhinolux Superior Paints, 2022).

The FGDs incorporated images of cool roofs. Visual cards covering elements of cost, durability, safety visual appeal, effectiveness in reducing heat and ease of installation were presented to participants. An interactive ranking exercise using stickers to assess priorities for features such as cost, durability, visual appeal, safety, ease of installation, and effectiveness in reducing heat, was conducted. Five stickers were ranked as the most important, and one sticker was ranked as the least important.

Discussions were recorded with the participant’s permission, and a notetaker was present to manually record observations and interactions.

### Data management and analysis

During data collection, participants were asked not to share personal information. All participants were de-identified during the transcription of the recordings and notes, and codes were assigned to them. All data was stored under password protection and was only available to key individuals on the research team.

The lead FGD facilitator conducted the translation and is fully conversant in English and isiZulu with extensive experience in transcription and translation. The facilitator is from USINGA and preserved the cultural and contextual meaning of the accounts. Emphasis was placed on the conceptual and cultural equivalence rather than literal linguistic correspondence. The coding and analysis were shared with the facilitator to ensure the analysis was accurate. Although back translation was not conducted, the translational and conceptual accuracy was enhanced through review and discussion of excerpts amongst the bilingual members of the research team while preserving the cultural and contextual accounts of participants.

The qualitative data collected from the FGDs were analyzed using inductive thematic analysis. An inductive, data-driven approach was employed to ensure that the themes emerged directly from participants’ narratives rather than being imposed by pre-existing theoretical frameworks. This approach aligns with the study’s exploratory aim to explore community participants’ perceptions and attitudes toward passive cooling interventions. The analysis process followed six iterative steps, as recommended by Braun and Clarke (2006). (1) The first stage involved immersion in the data through careful reading and re-reading of the verbatim transcripts from all FGDs. During this stage, the researcher noted initial impressions, recurring words or phrases, and key ideas that appeared significant to the research questions. Memos were created to capture early reflections and to summarize potential patterns across groups. (2) In the second stage, systematic manual coding was undertaken. Each transcript was reviewed line by line to identify meaningful units of text related to participants’ experiences of heat exposure, health impacts, coping strategies, and perceptions of the passive cooling intervention. Codes were kept as close to participants’ language as possible to preserve authenticity and reduce researcher bias. Both descriptive codes (e.g., “difficulty sleeping due to heat”) and interpretive codes (e.g., “climate-related anxiety”) were documented. Coding was primarily inductive but guided by the overall research aim and questions. To strengthen analytic rigor, two researchers (PN and NNR) independently reviewed and coded the transcripts before comparing interpretations and discussing emerging codes and themes. The differences in coding were resolved through discussion and consensus, with reference to the original transcripts where necessary. SN reviewed the final coding and analysis independent of PN and NNR. (3) Once the coding was completed, data were collated into similar or related codes and then into potential themes that represented broader conceptual categories. This process was supported by constant comparison, examining similarities and differences between participants’ accounts across the different focus groups. At this stage, visual mapping and thematic matrices were used to explore the relationships between codes and to identify emerging patterns of meaning. (4) The preliminary themes were then reviewed against the coded data and the entire dataset to ensure coherence, consistency, and representativeness. This step involved checking whether each theme accurately reflected participants’ experiences and whether there was sufficient data to support it. Overlapping or redundant themes were merged, while those lacking sufficient evidence were discarded or subsumed under broader categories. This review process also involved collaborative discussions among the research team to enhance the credibility and dependability of the findings. (5) The fifth step involved defining the essence of each theme and determining its scope and boundaries. Each theme was given a clear, concise name that encapsulated its core meaning and contribution to the overall narrative. Sub-themes were identified to represent more specific aspects of each main theme. (6) The final stage entailed weaving the themes and sub-themes into a coherent analytical narrative. Verbatim quotes were selected to illustrate each theme, ensuring that participants’ voices anchored the interpretation.

During the analytic process, recurring patterns and consistency across participant responses indicated that thematic saturation was achieved, as no substantially new themes emerged in the second focus group discussion (Braun and Clarke, 2021). However, given that only two FGDs were conducted, additional discussions may have yielded further nuanced or divergent perspectives, particularly across gender categories. Future studies should therefore consider conducting additional and gender-stratified FGDs to strengthen thematic depth and diversity of perspectives.

### Ethics

The study was approved by the Biomedical Research Ethics Committee of the University of KwaZulu-Natal (BREC/00008612/2025) and the Institutional Review Board (IRB) of the Harvard T.H. Chan School of Public Health (IRB25-0195). All participants provided written informed consent.

## Results

A total of 20 adults participated in the pilot study, with sociodemographic information on the participants presented in Table 1 below. The majority were female (*n* = 13; 65%), with a mean age of 39.9 years (Standard Deviation: 9.4 years), had completed high school (*n* = 10; 50%) but were unemployed (*n* = 11; 55%).

**FIGURE 1:**
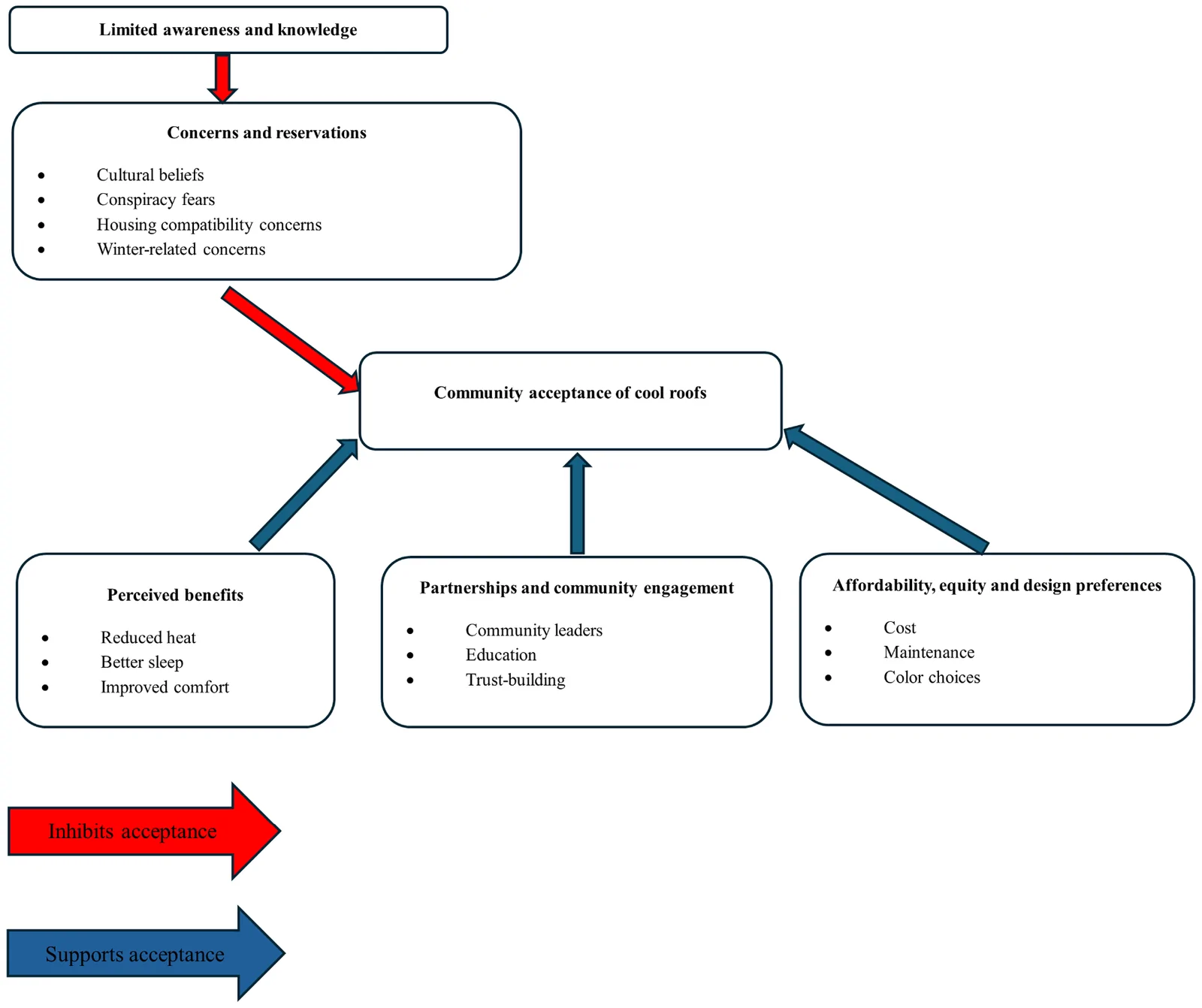
Relationship of themes that emerged from the focus group discussion on cool roofs.

The analysis of focus group discussions revealed six overarching themes that reflect community perceptions and their attitudes regarding the cool roof interventions. The discussion of themes highlights the interconnections between perceptions and attitudes toward cool roof interventions as reflected in Figure 1.

Themes that emerged around the cool roof interventions included (a) limited awareness and knowledge of cool roofs as a heat adaptation strategy, (b) perceived benefits and acceptance, (c) concerns and reservations, (d) affordability, and equity and (e) design preferences. The importance of partnerships and community engagement emerged as a final theme.

### Limited community awareness and knowledge of cool roofs as a heat adaptation strategy

Participants indicated that the cool roof concept was new to them. In FGD 1, when asked whether they had heard about the system before, participants collectively responded, *“No.”* Similarly, in FGD 2, the majority reported that this was their first time hearing about the cool roofs, as exemplified by Participant 1, who indicated that “*this is new to us.”* This suggests that knowledge of cool-roof technologies remains low at the community level.

One participant indicated having knowledge of rubber roofing, a common technique used for preventing water infiltration and resisting damage from extreme weather. They explained that:

“I’ve heard of something called rubber roofing – a black rubber layer added to the roof. I’m not sure if it serves the same purpose, but I know it exists.”

Participant 2, FGD2.

Another participant explained that they had come across a different strategy that focused on painting walls to reduce heat:

“I have heard about it [cool roofs] before, there was project that did not focus on the roofs, but they painted the walls as well because you can find that sun and heat come from the wall side, but the project did not pass.”

Participant 4, FGD1.

This indicates that although the specific technology is unfamiliar, the broader problem of heat exposure and the search for solutions are well understood. The lack of awareness and knowledge of the cooling roof system did not translate into resistance. Instead, participants demonstrated willingness to engage with the idea. For example, one of the participants indicated that:

“…We will have to see how these cool roofs work because we are not aware about them.”

“Participant 1, FGD1.

### Perceived benefits and acceptance

Participants reported overall positive attitudes toward the cool-roof concept, recognizing its potential to reduce indoor heat and improve comfort, especially for those in informal dwellings. The participants expressed positive sentiments, explaining that cool roofs will be particularly useful among individuals experiencing extreme indoor heat without access to cooling technologies, as illustrated by the following:

“I think this solution will help a lot especially since I live in a rental unit. These units retain a lot of heat… So this solution will be very helpful, even to the elderly people and children. Children really suffer from the heat.”

(Participant 3, FGD2).

“If this can lower indoor temperatures, I would welcome it greatly. The heat we endure inside our homes is intense and makes life very hard. This could help many people living in houses without fans or air conditioning.”

(Participant 2, FGD2).

“Once this is applied, houses will experience much less heat. This will help young children, older adults, and people who struggle with heat at night. It could also reduce the extreme fatigue of people living in hot homes “

(Participant 5, FGD2).

Participants viewed the intervention as both timely and necessary, given their lived experiences of excessive heat. Their acceptance is grounded in practical benefits, which include cooler indoor temperatures, better sleep, and protection for vulnerable groups. The enthusiasm of participants also reflects openness to innovation when interventions are perceived as directly improving comfort and health.

### Concerns and reservations

Despite the general positive attitudes towards the cool roof concept, some participants had cautious attitudes due to uncertainties about the project’s long-term effectiveness and compatibility with existing housing structures. For example, some participants noted that the roof concept may not be ideal for informal settlements:

“I hear what they are saying about roof but the thing is an informal settlement is made of something like a roof, so to me I do not see this paint achieving its goal, there should be another way for people residing in informal settlements because this would not work.”

(Participant 2, FGD1).

Similarly, concerns were raised regarding the effectiveness of the coating on houses that are double-storey or built using asbestos roofs:

“In Umlazi, many houses are double-storey RDP units. If this coating is applied on the top floor, will it also benefit the rooms downstairs?”

(Participant 3, FGD2 - Roofing section).

“Most houses in Umlazi have tiled or asbestos roofs, not just steel. Will the coating work on those as well?”

(Participant 4, FGD2 - Roofing section).

Other respondents also noted that there might be challenges to the adaptation of the cooling roofs regarding cultural beliefs and conspiracy theories as noted below.

“Since we are talking about colour change, elderly people believe that white roof is easy to be blown away by wind because they believe that ‘inkanyamba’ ^1^ is drawn by that colour. Maybe that could be an issue because of those beliefs.”

(Participant 2, FGD1).

Additionally, the influence of misinformation and distrust, particularly driven by social media narratives was highlighted as a potential barrier to community acceptance of the intervention. One participant explained:

“It will be hard to paint certain houses because of social media; there are many conspiracy theories on social media which lead to people not accepting help that will actually benefit them. They will be very wary; some will think that they will be infected by a certain disease. Some will say that the ANC is there to kill them with paint.”

(Participant 3, FGD1).

Community-level political tensions were highlighted as an additional factor that could undermine acceptance, with participants suggesting that opposition to local leadership structures may contribute to the spread of misinformation. As one participant explained:

“Another thing that might be a problem are political dynamics in the community; some people are against councillors, so they might spread conspiracies.”

(Participant 4, FGD1).

Beyond cultural beliefs, participants also raised practical concerns about seasonal performance. Participants expressed ambivalent views, indicating conditional acceptance alongside concerns about the implications of colder weather conditions. This ambivalence was often grounded in personal experiences and consideration of vulnerable individuals, such as elderly family members with heightened sensitivity to cold:

“I am 50–50 about it… I am just thinking of my mother as well because she has extreme sensitivity to cold, I am just wondering how she would view this… the researchers should try and research what they could do when it gets very cold in winter.”

(Participant 1, FGD1).

“There will be an approximate of 10% who will ask what is going to happen when it is in winter and cold, which means that we will have to adapt to more cold than what we are used to.”

(Participant 1, FGD1).

Overall, participants highlighted the need for contextually appropriate adaptation strategies, emphasizing that responses should account for seasonal variability and the specific needs of vulnerable groups. The fear of overcooling reflects practical lived experience in dwellings with poor insulation. It suggests that interventions must be climate-appropriate year-round, not only for peak summer temperatures. These views emphasize the importance of transparency, as well as pilot testing to evaluate the suitability of cool roof interventions during colder winter conditions, alongside community education prior to implementation.

### Affordability and equity

This theme highlights economic considerations that shape participants’ views about accessibility and fairness in implementing the intervention. The cost of the paint and its equitable distribution were major points of discussion. Most participants were concerned about the costs as illustrated below:

“R2000? That means no one would buy that paint… Maybe let us just say R800-R900, but you cannot say R2000”

(Participant 2, FGD1)^2^.

“The thing is most people are unemployed.”

(Participant 3, FGD1).

“Some people would rather suffer from heat because they would not want to pay.”

(Participant 2, FGD1).

Affordability emerged as a critical determinant of acceptance. Participants emphasized that equitable access is crucial for the intervention to succeed in low-income areas. Their suggested prices demonstrate a willingness to contribute modestly, but reinforce that subsidies or donor support would be necessary for broad adoption.

### Design preferences

Participants expressed strong views regarding the color of cool roof coatings, revealing a tension between thermal performance requirements and personal aesthetic values. The dominant preference was for color variety and choice, even though participants understood the functional rationale for white roofs. A key concern emerged regarding existing roof colors. One participant asked:

“So, if I have a green roof and I repaint using the cool roof paint, will it still be effective or I have to remove my paint first?”

(Participant 4, FGD 1).

Similarly, another participant asked:

“We have to ask if we would be able to paint with our own colours on top of the cool roof paints.”

(Participant 1, FGD 1).

As such, several participants explicitly called for a range of colors rather than a single option. As one participant noted:

*“…there should be a wide* var*iety of colors in the long run so that people can choose which one they want.”*

(Participant 1, FGD 1).

This preference emerged from an awareness that community members invest in harmonizing their home exteriors. The participant further explained:

“I think one of the biggest problems will be the color because whenever people are building their houses, they mix and match their colors. So, the white paint might be an issue.”

(Participant 1, FGD 1).

Another participant elaborated on this concern:

“I think some people might not like this project because it changes the color of the roof; some people painted their tiles in specific colors that they like. Changing it to a different colour like might be an issue, I think people would be receptive if they paint the inside, not the outside, I do not know if that is possible. Some people make sure their roofs match their walls, so maybe some people would not want this because of the colour”

(Participant 2, FGD 1).

This reveals that for most community members, roofs are not merely functional elements but are integrated into broader aesthetic schemes where color coordination between roofs, walls, and other exterior features is valued. Despite these preferences for variety, there was also pragmatic acceptance of white when its functional benefits were understood.

In addition to design preferences, few participants were also concerned about the durability of roofing as noted below:

“Okay so when we remove the paint you had installed because we are changing the roof types, will you come back and repaint or do I have to buy the paint myself? People will ask that question.”

(Participant 1, FGD1).

Such practical questions about durability and maintenance demonstrate a long-term perspective toward intervention sustainability.

### Importance of partnerships and community engagement

The participants consistently emphasized that for the project to be trusted, accepted, and successful, it must involve a deep and strategic partnership with the community and its existing structures. One way was to involve trusted community leaders in the project because of the power they hold in communities as trusted messengers. Some of the participants who were community leaders explained that:

“If my roof gets painted before the end of the year and it works, obviously I will tell my neighbor and my neighbor will want to buy it as well… by the time it is going to the government, everyone would already be wanting to have it because they know about it… Yes, because my neighbors will want it as well if they see that it is really working, so basically, I am the one who is selling the product “

(Participant 2, FGD1).

“I think the people would support this initiative if we have our roof painted as well, because we have to lead by example and we have to experience it first before endorsing it to other people.”

(Participant 3, FGD1).

Participants explicitly advised the researchers to integrate into existing formal, multi-stakeholder community platforms. These platforms will be important as they will provide a platform for researchers to present their ideas to the community:

“Also, we have war rooms and there is usually relevant stakeholders and government departments, and the community representing the voting districts. I think there should be someone from the project who will introduce this initiative might make things easier.”

(Participant 3, FGD1).

“Yes, all the stakeholders meet at the war room to discuss everything. USINGA should have a representative in every war room so they can present this project and also get a chance to meet different people from different voting districts, and they should attend the meetings regularly to report changes if there are any.”

(Participant 4, FGD1).

Participants also identified specific partners whose buy-in is crucial for addressing health-related concerns and legitimizing the project. For example, the Department of Health was considered essential:

“People from the department of health should be educated about this paint so that they might not blame every sickness or disease on it. A lot of people should be present and be made aware and educated about this paint, even social workers… I think department of health is the most important stakeholder in this case.”

(Participant 5, FGD1).

The community’s feedback extends far beyond simple reactions to the proposed cool roof technology. Rather than being passive recipients, the community had suggestions to offer regarding co-design, employment creation and scale-up. For example, the participants indicated that it was important to consult with the community regarding pricing of the cooling roofs:

“In terms of costs, I think they should consult with the community and hear what they have to say and come to an agreement.”

(Participant 3, FGD1).

The results also indicate that the participants were keen to know how the community was going to benefit in terms of job provision for local people to be involved in the implementation, as noted below:

“Some people might look into how they are going to benefit, there should be a Durban branch and maybe hire people, something like that… maybe you can pass that to the principal investigators of this project because we want to work.”

(Participant 3, FGD1).

## Discussion

In summary, the community participants appeared to have limited awareness and knowledge about cool roofs as a heat reduction intervention. Nevertheless, they had positive attitudes toward the intervention and perceived the possible benefits to the health of the community. Community members expressed concerns and reservations around cultural acceptability, affordability, and design preferences of the intervention in the community. Importantly, they agreed that for intervention implementation and sustainability, there had to be engagement and partnerships built with community members.

This exploration of community members’ perspectives and attitudes toward a cool roof intervention in a peri-urban setting in South Africa yielded important information that can inform the implementation of cool roofs for heat reduction in this setting and similar settings in the country and across Africa. In USINGA, the interviewed community members had limited prior knowledge and awareness of the cool roof concept, but showed a willingness to engage, which is very promising for intervention implementation. Community members perceived the benefits and advantages of cool roof interventions, particularly for high-risk groups within their community. Temperatures in their homes are very high, and due to the socio-economic challenges they face, they are unable to afford cooling methods such as air conditioners, which would be considered a luxury in this context (Statistics South Africa, 2018).

The literature reports that older people, the sick, and children are at the greatest risk for heat-related adverse health outcomes (Schlader et al., 2024; Azan et al., 2025). The lived experience of the USINGA community reflects their recognition of the adverse impact of high temperatures on these at-risk groups and the benefits of a cool roof intervention for such groupings within their community. Another important phenomenon they report on is the disruption of sleep patterns at night in this community due to high temperatures, an occurrence that has been documented in the literature. A previous meta-analysis reported an association of degraded sleep quality and quantity with rising outdoor and indoor temperatures globally (Chevance et al., 2024). Notably, the review found that the effect of temperature on sleep is greatest in the warmest months and affects the most vulnerable people in communities, particularly the elderly. Sleep pattern disturbances are known to lead to cardiovascular and metabolic diseases and increase stress, anxiety and depression (Shah et al., 2025). Therefore, heat exposure in our study context can only add to the health challenges experienced in this community with an existing high disease burden.

Nevertheless, despite the positive benefits noted by community members, they raised some concerns, which appear to be similar to those raised by the community in a study conducted by Barnighausen et al. (2025) in Burkina Faso. In the Burkina Faso study, participants were willing to accept the cool roofs, provided they reduced indoor heat and felt that local ownership and production were important to successful implementation. The cost of the cool roof was an important consideration for long-term sustainability. Similarly, amongst the USINGA participants, they believed that the cool roofs would be acceptable if they could lower indoor temperatures. As highlighted previously, they were concerned about sustainability and cost, and wanted the local community to be involved in the implementation of the intervention.

Community members were clearly concerned about the effectiveness of the cool roof paint, particularly in relation to housing structures. In USINGA informal dwellings are unlikely to have roofs similar to those of formal structures, and the concern raised about the application of cool roof paint is a valid one. Furthermore, several homes in the community have asbestos roofs, and concerns have been raised about the effectiveness of cool roof paint on this surface. However, recent research demonstrates that the cool roof intervention is effective in both informal and formal dwellings. A comparative study in Shanghai, China, also reported indoor temperature reductions in high-rise buildings (He et al., 2020). Maintaining indoor temperatures at levels that ensure a comfortable experience across different seasons for inhabitants is another concern for the community.

When introducing any community-based intervention, it is crucial to consider the cultural attitudes and beliefs of the target community. Failure to do so can halt and hinder implementation, even of the most beneficial of interventions. In the FGDs, community members raised concerns that the cool roof intervention might be misinterpreted as either a cause of bad weather or a tool used by certain political parties to kill people. With reference to being the cause of bad weather, participants made reference to “inkanyamba” who is supposed to be the deity of tornadoes and is attracted to white roofs. This perception will need to be explored further and addressed within the community prior to implementing the cool roof intervention. Similarly, aesthetic values were raised by the participants, indicating differences in preferences for roof colors. This highlights the need for proper community engagement prior to implementation of the intervention (Stover et al., 2024).

There is a high level of unemployment in Umlazi (Statistics South Africa, 2018). Hence, the cost of the cool roof intervention will be of great concern to the residents, as echoed by the community members in the FGDs. This may decrease uptake of the intervention. Furthermore, it is crucial that the most vulnerable individuals in this community can access the intervention, thereby preventing or reducing heat-related adverse health outcomes. Participants raised the possibility of subsidizing the intervention to aid uptake and sustainability. Incentives have been used in healthcare to increase uptake. However, these have only yielded short-term gains, with many being unable to sustain incentive uptake in the long-term following implementation (Saunders et al., 2025; Glanz et al., 2026).

Participants were clear that for the cool roof intervention to be successfully implemented and sustained, there must be extensive community engagement, consultation, and use of existing community platforms. The “war room” concept was introduced to KwaZulu-Natal as part of the Operation Sukuma Sakhe (OSS) project in 2009 to address socio-economic inequity and improve service delivery across all sectors in the province (Mkhize, 2023). The “war room” operates at the ward level, which is the lowest level of government structure in South Africa, and is led by the ward councilor, who is elected by the community. Included in the “war room” are community representatives and local government officials. The “war room” should meet weekly to discuss and address local community challenges. Participants highlighted the importance of involving local “war room” members in the project to ensure successful implementation and sustainability.

The strength of this study was in the willing participation of representatives from the USINGA community. These are respected community members who were willing to participate in the study and share their perspectives. A limitation was in the gender imbalance of the participants (more females than males), and hence, we may not have obtained a true representation of male perspectives on the issue of cool roofs. For example, male participants may have offered different perspectives on housing modifications, as they have decision-making power in most African communities. Therefore, future studies should consider gender-stratified focus group discussions to ensure more balanced representation. An additional limitation was in the number of FGDs conducted (*n* = 2). This limitation also has implications for the transferability of the findings, as communities vary widely in terms of socio-cultural beliefs, climate, politics, housing, poverty, and access to services. Nevertheless, broader themes identified in this study, including the importance of affordability, trust, community engagement, and culturally sensitive implementation, may resonate across comparable low-resource settings.

These findings suggest that cool roofs can be a highly relevant, pro-poor climate adaptation measure in other African countries, especially those with hot climates, rapid urbanization, extensive informal settlements, and low access to air conditioning. However, as in South Africa, success elsewhere in Africa will depend on tailoring interventions to local roofing materials and settlement types, and on addressing cultural meanings and political sensitivities. Formative research similar to this study is warranted in other African settings to understand local perceptions, priorities, and potential barriers. Implementation research is needed in Umlazi and elsewhere to assess the feasibility, acceptability, and effectiveness of this intervention, as well as to examine effective delivery models, financing approaches, and community engagement strategies for sustainable scale-up.

## Conclusion

This pilot study to better understand community awareness and knowledge, perceptions, and attitudes toward the concept of cool roofs provided valuable information to inform a larger study on the overall aim. While participants had limited awareness and knowledge of the cool roof concept, they were willing to engage and provided suggestions on how to implement and sustain such an initiative. Further, they identified possible challenges that would need to be addressed to ensure the successful implementation of the intervention. Overall, there was a positive attitude toward the intervention, which should bode well for future implementation. As the next steps in this process over the next year, meetings will be held to inform stakeholders, including community members, local leaders, and the Department of Health, about the cool roof intervention. Further focus group discussions and key informant interviews will be held with community members to establish their awareness, perceptions and attitudes toward the cool roof intervention. Thereafter, a pilot study will be conducted with a sample of willing households to assess the feasibility of implementing the cool roof intervention and its impact on indoor household temperatures and participants’ health.

## Figures and Tables

**TABLE 1:** Sociodemographic characteristics of study participants (*n* = 20).

Characteristics	*N* (%)
**Gender**	
Male	7.0 (35.0)
Female	13.0 (65.0)
**Mean age (standard deviation, in years)**	
Overall	39.9 (9.4)
Male	39.0 (7.9)
Female	38.9 (10.4)
**Highest education level**	
Post-matric education	7 0.0 (35.0)
Completed matric	10.0 (50.0)
High school but no matric	3.0 (15.0)
**Employment status**	
Formal sector employed	3.0 (15.0)
Informal sector employed	6.0 (30.0)
Unemployed	11.0 (55.0)

## Data Availability

The raw data supporting the conclusions of this article will be made available by the authors, without undue reservation.

## Footnotes

1 This refers to what is understood to be a deity of tornadoes and severe rains, appearing in the form of a black serpent in Southern African folklore, which is supposed to rise from the deep waters of the Howick Falls, a waterfall located in the KwaZulu-Natal midlands.

2 1 US dollar = 16.5 South African rands.
